# Flip-flop phenomenon on dual SSTR PET and amino acid PET in a case of recurrent meningioma with malignant transformation

**DOI:** 10.1007/s00259-024-06998-y

**Published:** 2024-11-26

**Authors:** Adrien Holzgreve, Patrick N. Harter, Robert Forbrig, Stefanie Quach, Niklas Thon, Christian Schichor, Joerg-Christian Tonn, Maximilian Niyazi, Matthias Brendel, Louisa von Baumgarten, Nathalie L. Albert

**Affiliations:** 1 Department of Nuclear Medicine, LMU University Hospital, LMU Munich, Marchioninistr. 15, 81377, Munich, Germany; 2 Ahmanson Translational Theranostics Division, David Geffen School of Medicine at UCLA, Los Angeles, CA USA; 3 Institute of Neuropathology, Faculty of Medicine, LMU Munich, Munich, Germany; 4 German Cancer Consortium (DKTK), partner site Munich, a partnership between DKFZ and Ludwig-Maximilians-Universität München (LMU), Munich, Germany; 5 Bayerisches Zentrum für Krebsforschung (BZKF), partner site Munich, Munich, Germany; 6 Institute of Neuroradiology, LMU University Hospital, LMU Munich, Munich, Germany; 7 Department of Neurosurgery, LMU University Hospital, LMU Munich, Munich, Germany; 8 Department of Radiation Oncology, University Hospital Tübingen, Tübingen, Germany; 9 Department of Radiation Oncology, LMU University Hospital, LMU Munich, Munich, Germany; 10 Center for Neuro-Oncology, Comprehensive Cancer Center Tübingen-Stuttgart, University Hospital Tübingen, Tübingen, Germany; 11 German Cancer Consortium (DKTK), partner site Tübingen, a partnership between DKFZ and University Hospital Tübingen, Tübingen, Germany; 12 DZNE– German Center for Neurodegenerative Diseases, Munich, Germany; 13 Munich Cluster for Systems Neurology (SyNergy), University of Munich, Munich, Germany

**Keywords:** [^18^F]FET, [^18^F]SiTATE, Somatostatin receptor, PET imaging, Differential diagnosis, Dedifferentiation

Amino acid PET is used for glioma imaging but has no established role in meningioma [1, 2]. We present a “flip-flop” constellation on SSTR and amino acid PET in meningioma that enabled to detect unrecognized malignant tumor tissue. A 65-year-old patient in continuous clinical follow-up presented with a new contrast-enhancing lesion on MRI in the left cerebral peduncle (red arrows), 5 years after radiotherapy of a left temporal suspected low-grade meningioma, and 1 year after its resection (revealing atypical meningioma CNS WHO grade 2). The patient received ongoing everolimus/octreotide for dural tumor remnants (green arrows). MRI findings were suggestive of reactive changes but could not exclude vital tumor. SSTR-targeted PET/CT with 187 MBq [^18^F]SiTATE showed markedly increased SSTR-expression at the known residue, whereas the adjacent new lesion only showed low tracer uptake, suggesting radiation necrosis [3]. Due to uncommon late-onset after radiotherapy, additional amino acid PET with 173 MBq [^18^F]FET was performed. In contrast to SSTR-PET, [^18^F]FET-PET displayed only minor uptake in the known meningioma residue; and while the time-activity curves were continuously increasing in the dynamic analysis, the new lesion showed markedly increased [^18^F]FET uptake, typical for malignant tumor tissue. Taken together, the findings were suggestive for meningioma recurrence with signs of dedifferentiation and malignant transformation. Stereotactic biopsy revealed malignant tumor tissue but was inconclusive regarding the tumor type. Eventually, surgical resection of the new lesion revealed malignant meningioma, now classified as CNS WHO grade 3, including homozygote CDKN2A/B deletion in the DNA-methylation profile [4], which was not present in the initial CNS WHO grade 2 tumor. Additional amino acid PET imaging in meningioma may help to identify metabolically active dedifferentiated tumor tissue in cases with equivocal previous imaging findings.

**Figure 1:**
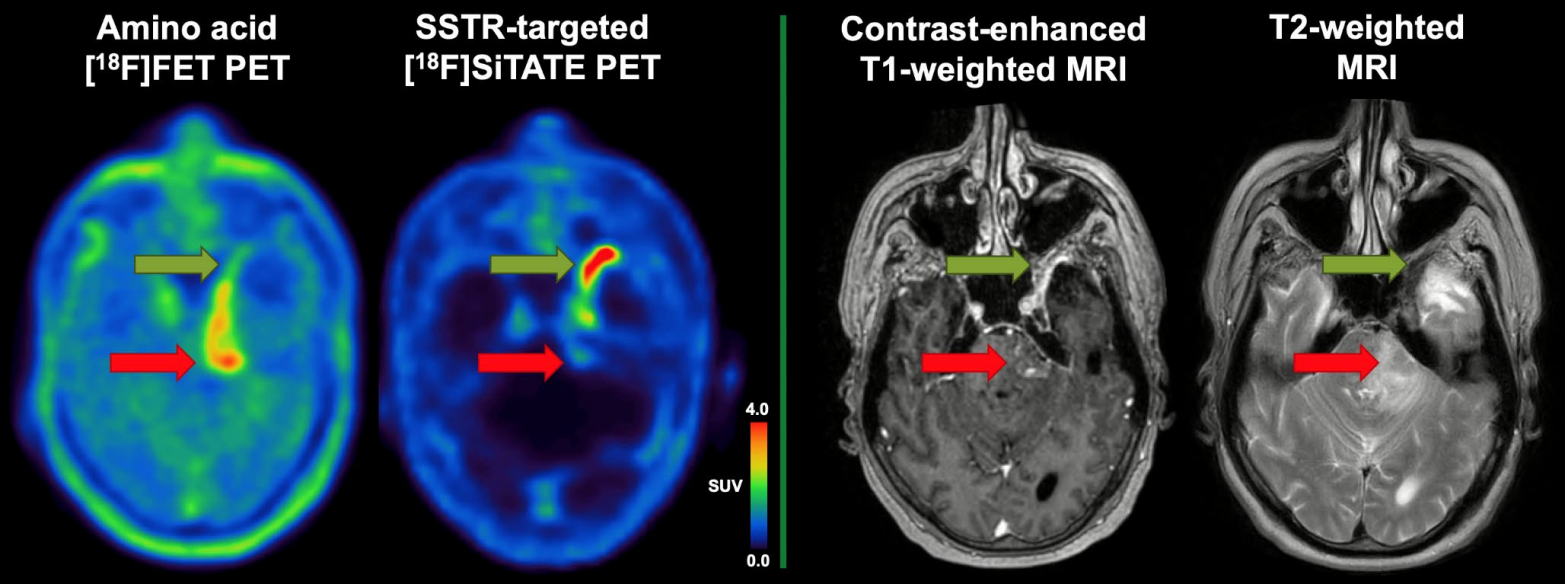

## Data Availability

The datasets used and/or analyzed during the current study are presented in the manuscript.

## References

[1] Albert NL, Preusser M, Traub-Weidinger T, Tolboom N, Law I, Palmer JD, et al. Joint EANM/EANO/RANO/SNMMI practice guideline/procedure standards for diagnostics and therapy (theranostics) of meningiomas using radiolabeled somatostatin receptor ligands: version 1.0. Eur J Nucl Med Mol Imaging. 2024. 10.1007/s00259-024-06783-x. 38898354 10.1007/s00259-024-06783-x PMC11445317

[2] Holzgreve A, Albert NL, Galldiks N, Suchorska B. Use of PET Imaging in Neuro-Oncological surgery. Cancers (Basel). 2021;13. 10.3390/cancers13092093. 10.3390/cancers13092093 PMC8123649 33926002

[3] Müller KJ, Biczok A, Schichor C, von Baumgarten L, Albert NL. The value of [(18)F]FET PET and somatostatin receptor imaging for differentiating pseudoprogression in residual meningioma. Eur J Nucl Med Mol Imaging. 2024;51:1194–6. 10.1007/s00259-023-06479-8. 37897618 10.1007/s00259-023-06479-8 PMC10881591

[4] Sahm F, Aldape KD, Brastianos PK, Brat DJ, Dahiya S, von Deimling A, et al. cIMPACT-NOW update 8: clarifications on molecular risk parameters and recommendations for WHO grading of meningiomas. Neuro Oncol. 2024. 10.1093/neuonc/noae170. 39212325 10.1093/neuonc/noae170 PMC11812049

